# Editorial

**Published:** 1869-02

**Authors:** 


### Editorial.

ANNUAL MEETING OF THE ILLINOIS STATE MEDICAL SOCIETY.—The next Annual Meeting of the Illinois State Medical Society will be held in Chicago, commencing on the *third Tuesday* in May, 1869. We expect a full and interesting meeting; and hope the profession, in every part of the State, will be represented.

AMERICAN MEDICAL ASSOCIATION.—The next Annual Meeting of the American Medical Association will be held in New Orleans, on the *first Tuesday* in May, 1869. As this is the first meeting in the South for several years, it is very desirable that it should be well attended. A full delegation from the Northwest will be most cordially received by the Profession in New Orleans, and will do much, not only to advance the strictly professional and scientific interests of all sections, but will also aid in completing the restoration of cordial feelings of brotherhood throughout the land.

SPRING AND SUMMER MEDICAL INSTRUCTION IN CHICAGO.—The Rush Medical College closes its usual short Annual Term on Wednesday, February 3d (before this number of the EXAMINER will reach its readers), by the ordinary exercises of public commencement, and a meeting of its Alumni. By a circular that accompanies this number of the EXAMINER, it will be seen that arrangements have been made for a Spring Term of instruction, in connection with that College, extending from

March to July. The instruction to be given in the Spring Course is to be mostly by Young Men of talent and enterprise, not members of the Faculty of the Rush College, but connected with the Public Hospitals and Dispensaries of the City. For details, see the circular.

The regular Annual Lecture Term of the Chicago Medical College does not close until the *fourth Tuesday* in March. A Summer Course of instruction, commencing on the first Monday in April, and ending on the first of July, constitutes a permanent part of the organization of this College; and is free to all medical students who become regular Matriculants of this College. It will be seen that no City in the Country affords better facilities for the study of Medicine, in all its departments, both Winter and Summer, than Chicago.

---

Honorary Degrees.—We see it stated in the daily papers, that "the Faculty of Lake Forest University, at a recent meeting, conferred the honorary degree, Professor of Ophthalmic and Aural Science, upon Dr. J. S. Hildreth, of this city." This seems to be a novel "*Degree.*" We had supposed that a professorship involved an appointment, either active or honorary, in some institution. But this action of the Board of Trustees (not Faculty, for it has none) of Lake Forest University seems to confer a professorship on a gentleman, not only without an appointment, but without any such chair in the institution.

---

Money Receipts to Jan. 21, 1869.—Drs. W. H. Baxter, $1; James Miner, 3; J. Quirck, 6; Thos. S. Parr, 3; O. W. Sadler, 3; J. R. Flood, 3; C. G. Reim, 3; S. M. Pegram, 6; J. W. Barlow, 9; Henry Jones, 1; J. Woodworth, 2; Geo. McPherson, 6; J. F. Hopkins, 6; R. S. Lewis, 3; E. P. Cook, 3; Elmer, F. Clapp, 3; David Newell, 6; Asahel Clark, 3; A. S. Holland, 6; L. D. Payne, 4.75; J. S. Lawrence, 1; P. Eppler, 4; C. K. Clark, 3; J. P. Johnston, 3; V. Clarence Price, 3; I. N. Bishop, 5; G. S. Thomas, 3; W. H. Price, 3; W. W. Bonnell, 6; G. H. Fuller, 1.50; Levi Day, 3; A. Grættinger, 6; W. L. Kreider, 3; H. C. Miner, 6; D. J. Hussey, 4; M. W. Seaman, 5; Wm. Martin, 1.25; P. M. Cleary, 4; E. V. Dale, 3; Wood & Curtiss, 3; E. Andrews, 5; Thos. A. Clark, 6; Lyman J. Barrows, 3.25; Wm. F. Cady, 5; E. B. Wolcott, 6; C. Shumway, 6; Aug. Rhoads, 3; D. O. Crist, 2.50; H. K. Deene, 5; T. D. Fisher, 3; W. J. Johnson, 6; F. K. Bailey, 1; L. Tibbets, 2; A. M. Maxwell, 3.

VICK'S FLORAL GUIDE FOR 1869.—The first edition of one hundred thousand of Vick's illustrated catalogue of seeds and guide in the flower garden is now published. It makes a work of 100 pages, beautifully illustrated, with about 150 Fine wood engravings of flowers and vegetables, and an elegant colored plate, a boquet of flowers.

It is the most beautiful, as well as the most instructive Floral Guide published, giving plain and thorough directions for the culture of flowers and vegetables.

The Floral Guide is published for the benefit of my customers, to whom it is sent free without application, but will be forwarded to all who apply by mail, for Ten Cents, which is not half the cost. Address JAMES VICK, Rochester, N.Y.

